# The optimal intravesical maintenance chemotherapy scheme for the intermediate-risk group non-muscle-invasive bladder cancer

**DOI:** 10.1186/s12885-023-11523-9

**Published:** 2023-10-23

**Authors:** Jian-Xin Chen, Wen-Ting Huang, Qing-Yun Zhang, Cheng-En Deng, Jue-ling Wei, Yuan-Liang Xie, Rui Lin, Guan-Zheng Feng, Guang-Lin Yang, Jun Long, Hao-Yuan Lu, Zeng-nan Mo

**Affiliations:** 1 Institute of Urology and Nephrology, First Affiliated Hospital of Guangxi Medical University, Nanning, Guangxi 530021 China; 2 Department of Urology, Affiliated Tumour Hospital of Guangxi Medical University, Nanning, Guangxi 530021 China; 3 Center for Genomic and Personalized Medicine, Guangxi key Laboratory for Genomic and Personalized Medicine, Guangxi Collaborative Innovation Center for Genomic and Personalized Medicine, Guangxi Medical University, Nanning, Guangxi 530021 China; 4 Department of Nursing, Guangxi Health Science College, Nanning, Guangxi 530021 China

**Keywords:** Non-muscle-invasive Bladder cancer, Intravesical chemotherapy, Pirrubicin, Tumor recurrence

## Abstract

**Objective:**

Although the current European Association of Urology(EAU) guideline recommends that patients with intermediate-risk non-muscle-invasive bladder cancer (NMIBC) should accept intravesical chemotherapy or Calmette-Guerin (BCG) for no more than one year after transurethral resection of bladder tumor(TURBT), there is no consensus on the optimal duration of chemotherapy. Hence, we explored the optimal duration of maintenance intravesical chemotherapy in patients with intermediate-risk NMIBC.

**Subjects and Methods:**

This was a real-world single-center retrospective cohort study. In total 158 patients with pathologically confirmed intermediate-risk NMIBC were included, who were divided into 4 subgroups based on the number of instillations given. We used Cox regression analysis and survival analysis chart to explore the 3-yr recurrence outcomes of tumor.The optimal duration was determined by receive operating characteristic curve (ROC).

**Results:**

The median follow-up was 5.2 years. Compared with instillation for 1–2 months, the Hazard Ratios(HR) values of instillation for less than 1 month, maintenance instillation for 3–6 months and > 6 months were 3.57、1.57 and 0.22(95% CI 1.27–12.41;0.26–9.28;0.07–0.80, P = 0.03;0.62;0.02, respectively). We found a significant improvement in 3-yr relapse-free survival in intermediate-risk NMIBC patients who maintained intravesical instillation chemotherapy for longer than 6 months, and the best benefit was achieved with 10.5 months of maintenance chemotherapy by ROC.

**Conclusions:**

In our scheme, the optimal duration of intravesical instillation with pirrubicin is 10.5 months. This new understanding provides valuable experience for the precise medical treatment model of intermediate-risk NMIBC.

## Background

Bladder cancer is the sixth most common cancer in the world [1, 2]. Approximately 75% of bladder cancer patients present with non–muscle-invasive bladder cancer (NMIBC) [3, 4]. The 5-year recurrence rate and progression rate of NMIBC are 50–70% and 10–30%, respectively [5]. In order to reduce the likelihood of recurrence and progression, both the American Urological Association(AUA) and the European Association of Urology(EAU) guideline panels recommend intravesical chemotherapy after completed transurethral resection of bladder tumor(TURBT) [6, 7]. According to the EAU guidelines, NMIBC can be classified into low-risk, intermediate-risk, and high-risk ( including highest-risk) groups based on their clinical and pathological characteristics. For patients with low-risk recurrence in the past, it is recommended to receive one immediate instillation of intravesical chemotherapy immediately after TURBT [6]. For other patients with intermediate-risk tumors, either one-year full dose of bacillus Calmette-Guerin (BCG) or intravesical chemotherapy up to 1-yr is recommended [6].

Intravesical instillation of chemotherapy is an acceptable alternative method to BCG intravesical immunotherapy [8]. The selection of drugs for intravesical chemotherapy, such as adriamycin and mitomycin C, brings about the difference of chemotherapy scheme. Moreover, there is no consensus regarding the optimal duration or schedule of maintenance intravesical chemotherapy. Consequently, optimal decision-making for intravesical chemotherapy remains challenging, especially for patients with intermediate-risk disease, the largest proportion among NMIBC patients. The aim of this research was to determine the 3-yr relapse-free survival outcomes of intermediate-risk NMIBC patients who received different intravesical chemotherapy duration with pirarubicin. We tried to explore the optimal duration for intravesical maintenance chemotherapy in a cohort study by ROC curve.

## Method

### Cohort population and inclusion/exclusion criteria

This was a real-world single-center, retrospective cohort study based on southern China population. The inclusion criteria was patients with histological confirmed NMIBC. A total of 435 patients were enroled from Guangxi Medical University Cancer Hospital between July 2013 and December 2018. The risk of tumor recurrence was stratified according to the European Association of Urology guidelines on NMIBC updated in 2013 [9].

Exclusion criteria included the low-risk group NMIBC (Primary, solitary, Low Grade/Grade 1, diameter < 3 cm, no Carcinoma in situ) and high-risk group NMIBC(T1、High Grade/Grade 3、carcinoma in situ、multiple and recurrent and diameter > 3 cm TaG1/ G2 tumours (all these conditions must be presented) ). Other exclusion criteria included previous BCG or other intravesical chemotherapy in the preceding 12 months, previous radiotherapy, systemic chemotherapy or suffered from other cancers.

After subtyping according to EAU2013 guidelines, we first excluded 12 patients who met other exclusion criteria. Then we excluded 121 patients in the low risk recurrence group and 144 patients in the high risk recurrence group, and finally included 158 patients in the intermediate risk group in our research.

### Treatment protocol

According to EAU guidelines, these intermediate-risk NMIBC patients received a single immediate postoperative intravesical instillation of pirarubicin (30 mg diluted in 50mL saline) for 30 min [10] after completed transurethral resection of the bladder tumor(TURBT). In the following 8 weeks, additional instillations of pirarubicin(30 mg diluted in 50ml saline) were given once a week.Then, we maintained intravesical instillation using the same dose of the same drug once a month up to one year [11]. However, due to poor compliance or other reasons, they did not receive instillation chemotherapy as required by us. We recorded their instillation number and time in detail during follow-up and divided them into each instillation subgroup for analysis.

The case group was defined as intermediate risk NMIBC patients with recurrence within 3 years after Transurethral resection of bladder tumor(TURBT), and the control group was defined as intermediate risk NMIBC patients with no recurrence within 3 years.

After TURBT, 0–5 cycles of intravesical instillation was defined as the incompleted instillation group (equivalent to no instillation or instillation less than 1 month), 6–9 cycles of intravesical instillation was defined as the standard instillation group (equivalent to instillation for 1–2 months), 10–13 cycles (equivalent to instillation for 3–6 months) and > 13 cycles (equivalent to instillation for more than 6 months) were defined as the maintenance instillation group10-13 and maintenance instillation group > 13.

All patients underwent cytology and cystoscopy every 3 months for the first 2-years and 6-monthly thereafter. Patients with no recurrence for 3 years were examined as

the last follow-up. The main end point was the 3-year recurrence-free survival rate.

All data were collected by technicians from the medical records management office and these people were blinded to the purpose of the study. In case of missing or conflicting data, patient records were reviewed by independent quality control personnel.

### Statistical analysis

**Fig. 1:**
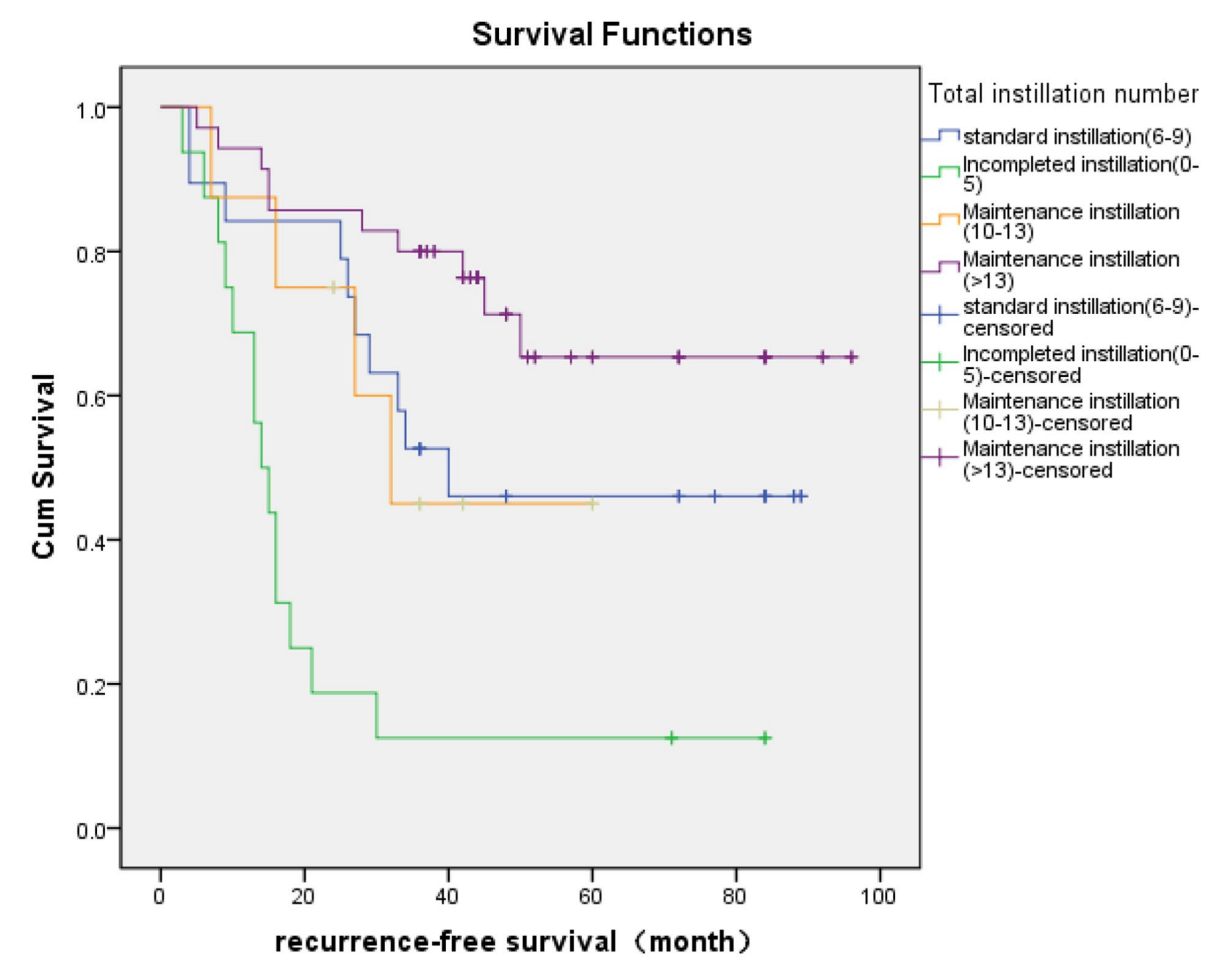
Kaplan-Meier curve for RFS stratified by total instillation number of intermediate-risk NMIBC.

**Fig. 2:**
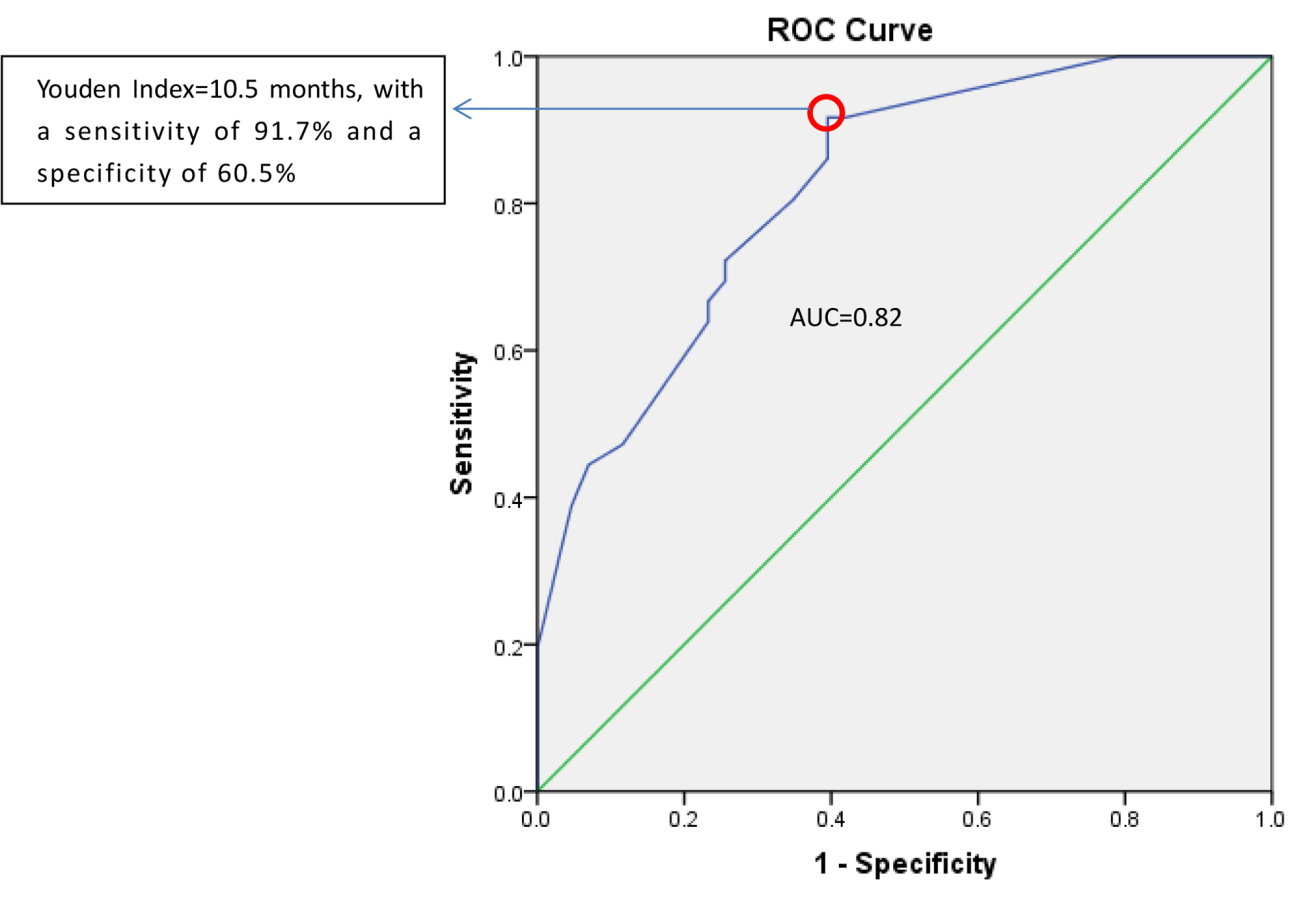
Total instillation months receiver operating characteristic curve(ROC) for intermediate risk NMIBC recurrence

All the analyses were performed with SPSS software, version 22.0. P value of statistical significance was set at P<0.05. Independent sample T-test, Pearson Chi-square test and Fisher’s exact test were utilized to analyze the parameter distribution(Table 1). Univariate Cox regression analysis identified significant variables associated with recurrence, and Multivariate Cox proportional hazard model was used to determine whether the total instillation number was an independent prognostic factor after adjusting for other potential risk parameters(Table 2).The relapse-free survival(RFS) for intermediate risk NMIBC stratified by total instillation number was estimated using the Kaplan–Meier method(Fig. 1). The optimal instillation month for intermediate risk NMIBC was evaluated by ROC curve(Fig. 2).

## Results

### Characteristics of study population

A total of 158 participants, including 136 males and 22 females, were enrolled in our cohort (Table 1). The median follow-up was 5.2 years. Although the average age, the number of tumors and the diameter of tumors in the case group were slightly higher than those in the control group (61.6 vs. 56.1, 2.75 vs. 2.30, 2.80 vs. 2.35, respectively),the BMI and previous recurrence rate in the case group were lower than those in the control group (21.9 VS 22.8, 44.4% vs. 53.5%, respectively).There was no significant difference in gender, age, BMI, number of tumours、tumour diameter and prior recurrence between the two groups.

The number of patients with incompleted instillation (immediate postoperative + weekly ×0–4 times) was 28(38.9%), while that of the control group was only 4(4.6%). In addition, most of these people (27/ 32) received only 0–1 intravesical instillation(not shown in the table). The number of standard instillation (immediate postoperative + weekly ×5–8 times) was equal between the two groups, with 20 people in both groups. The proportion of maintenance instillation in the control group(7%+65.1%) was significantly higher than that in the case group(13.9%+19.4%). The average total instillation numbers of the two groups were 7.33 versus 16.56.

**Table 1:** Baseline characteristics of patients with intermediate-risk NMIBC

Variable	Case (n = 72)	Control (n = 86)	P-value
Age			0.11
18–49 ≥ 50 Mean ± SD	12(16.7%) 60(83.3%) 61.61 ± 13.20	28(22.2%) 58(77.8%) 56.09 ± 12.67	
Gender			0.98
Female Male	10(13.8%) 62(86.2%)	12(11.6%) 74(88.4%)	
BMI	21.19 ± 6.09	22.85 ± 5.37	0.20
Number of tumours			0.27
Single ≥ 2 Mean ± SD	22(30.6%) 50(69.4%) 2.75 ± 2.41	34(39.5%) 52(60.5%) 2.30 ± 3.46	
Tumour diameter (cm)			0.37
< 3 ≥ 3 Mean ± SD	38(52.8%) 34(47.2%) 2.80 ± 1.51	54(62.8%) 32(37.2%) 2.35 ± 1.21	
Prior recurrence rate	44.4%	53.5%	0.42
Primary recurrence	40 32	40 46	
Total instilation number			P < 0.001
Incomplete instillation(0–5) Standard instillation(6–9) Maintenance instillation(9–13) Maintenance instillation(> 13) Mean ± SD	28(38.9%) 20(27.8%) 10(13.9%) 14(19.4%) 7.33 ± 6.64	4(4.6%) 20(23.2%) 6(7.0%) 56(65.1%) 16.56 ± 8.07	

### Cox regression for potential risk factors of moderate risk NMIBC

According to the guidelines of the European Organization for Research and Treatment of Cancer(EORTC) [8], the recurrence prediction model of NMIBC included six risk factors. Since high risk factors such as CIS (carcinoma in situ), HighGrade and T1 had been excluded from the cohort, we used univariate cox regression model to calculate the Hazzrd Ratios(HR) for NMIBC recurrence. HRs of tumor number ≥ 2, tumor diameter > 3 cm, and previous recurrence were 1.70,1.51 and 0.69(95%CI 0.65–4.40;0.61–3.71;0.28–1.69, P = 0.27;0.37;0.42 respectively).

Similarly, taking the standard instillation group as a reference, HRs of incompleted instillation group, maintenance instillation group 9–13 and maintenance instillation group > 13 were 3.40, 1.68 and 0.27(95% CI 1.12–12.03; 0.31–8.92 and 0.10–0.83, P = 0.03;0.55 and 0.02, respectively). Afterwards, Multivariate Cox proportional hazard model revealed that total instillation number (p = 0.001) was an independent prognostic factor for the recurrence of intermediate risk NMIBC. Compared with the standard instillation group, the HR values of incompleted instillation group, maintenance instillation group 9–13 and maintenance instillation group > 13 were 3.57、1.57 and 0.22(95% CI 1.27–12.41;0.26–9.28;0.07–0.80, P = 0.03;0.62;0.02, respectively)after adjusting for other potential risk factors(Table 2).

**Table 2:** Univariate and Multivariate Cox regression analysis for 3-yr tumor recurrence in patients with intermediate risk NMIBC.

Variable	HR (95% CI)	p value	aHR (95% CI)	p value
Number of tumours		0.27		0.31
Single ≥ 2	1 1.70(0.65–4.40)		1	
Tumour diameter (cm)		0.37		0.44
< 3 ≥ 3	1 1.51(0.61–3.71)		1 1.65(0.47–5.84)	
Prior recurrence rate		0.42		0.46
Primary recurrence	1 0.69(0.28–1.69)		1 1.61 (0.46–5.70)	
Total instillation number		< 0.001		< 0.001
Incompleted instillation(0–5)	3.40(1.12–12.03)	0.03	3.57(1.27–12.41)	0.03
Standard instillation (6–9)	1		1	
Maintenance instillation (10–13) Maintenance instillation (> 13)	1.68(0.31–8.92) 0.27(0.10–0.83)	0.55 0.02	1.57(0.26–9.28) 0.22(0.07–0.80)	0.62 0.02

### Kaplan-Meier survival curve showed a significantly higher RFS in the maintenance instillation group of NMIBC patients

Kaplan-Meier curve revealed that incompleted instillation(0–5) was related with a poor recurrence-free survival in intermediate risk NMIBC patients. However, the maintenance instillation (> 13) group had a significantly higher RFS compared with standard instillation(6–9) (Fig. 1).

### Total instillation month of 10.5 was a fairly diagnostic index for 3-yr recurrence of intermediate risk NMIBC patients

The ROC curve for 3-yr recurrence of intermediate risk NMIBC which was generated from the total instillation months, had an AUC of 0.822 (95% CI = 0.759–0.885) after correction for in-sample optimism by cross-validation. It suggested that total instillation month was a good discriminator for recurrence.The cut-off point (Y ouden Index)was 10.5 months, with a sensitivity of 91.7% and a specificity of 60.5% (Fig. 2).

## Discussions

Intravesical chemotherapy is recognized as an efficient therapeutic option for the prevention of recurrence in low or intermediate recurrence-risk patients, even though there is no agreement regarding a standardized protocol for intravesical chemotherapy [12]. EAU and AUA guidelines recommend that maintenance chemotherapy for a maximum of 1-yr could further reduce the recurrence rate of patients with intermediate risk NMIBC. So as to avoid the higher toxicity of BCG [13]. But there are still some scholars doubted the effectiveness of maintenance chemotherapy [14].

Thus, we need answers to the following questions: after early immediate instillation and a short-term weekly induction schedule, can additional maintenance intravesical chemotherapy further reduce the recurrence rate in intermediate-risk patients? Is there an optimal maintenance regimen?

Only a few large sample studies have explored this question. Koga et al. [11] in a prospective randomized trial compared 3-month vs. 12-month adjuvant therapy in patients who received early epirubicin(30 mg/30 mL). The authors reported a significant increase in RFR at 3 years in the long-term treatment group (31.5% versus 13% ,p = 0.005). Perhaps the suboptimal dose of epirubicin was a potential bias that could explain the better efficacy in the long-term maintenance.Friedrich et al [15] reported 3 years of maintenance therapy with mitomycin resulted in a 62% lower risk for recurrence than 6 weeks of induction therapy alone. In that study, immediate instillation was not given. Moreover, only 53% of the patients completed ≥ 1-yr of maintenance, raising doubts over whether a shorter period of adjuvant therapy could be adequate.

In contrast, a recent systematic review of maintenance intravesical chemotherapy of NMIBC declared that 13 of 16 RCTs reported no significant improvement in recurrence for patients receiving maintenance chemotherapy [14]. One of these RCTs reported by Vincenzo Serretta [16] in 2018 stated that 1-year maintenance after early adjuvant intravesical chemotherapy had a limited effect on preventing recurrence of intermediate-risk NMIBC. 482 Nmibc patients were randomized between a 6-week induction cycle and the induction cycle plus maintenance with 10 monthly instillations. Their results showed no significant difference in the non-recurrence rate (RFS) between the two groups (P > 0.05). However, their stratification criteria for NMIBC were based on EAU guidelines updated in 2002 [17]. But according to EAU guidelines up dated in 2013 [9], all T1 stage patients should be included in high-risk group rather than intermediate-risk group. Among the included cases, 308 of 482 were T1 stage. Such a high proportion of confounding factors leaded to a great deviation in the statistical results and few reliable conclusions.

Above all, firstly, the authors of previous studies lacked a comprehensive understanding of stratification by the risk factors of NMIBC. People with high risk factors such as T1/G3 were mistakenly included in intermediate risk group. Secondly, there were divergences between the AUA guidelines and EAU guidelines on the inclusion criteria of intermediate risk NMIBC [12, 13]. The above reasons leaded to inconsistent or even contradictory results of maintenance instillation chemotherapy.

Here we emphasize that we should re-evaluate the effect of maintenance instillation chemotherapy on reducing the recurrence of NMIBC in intermediate-risk groups according to the updated EAU guidelines [6].

Thus, we performed a cohort study to verify the oncologic outcomes of intravesical chemotherapy in patients with intermediate-risk NMIBC. This research led to several interesting findings.

Firstly, the average age of the case group is higher. It might be due to the poor mobility and poor compliance of elderly patients, resulting in a decreased number of instillation chemotherapy.

Secondly, the cox regression model revealed that only the total instillation number of intravesical chemotherapy was an independent risk factor for tumor recurrence (P < 0.001). The study [8] found that six factors(carcinoma in situ, HighGrade, T1 ,tumor number ≥ 2, tumor diameter > 3 cm, and previous recurrence) can predict tumor recurrence included all NMIBC patients (that is, low, intermediate, and high recurrence risk), while our study only included intermediate NMIBC recurrence risk patients. Moreover, the treatment plan of that study is BCG intravesical instillation for 1–3 years, not intravesical chemotherapy. Thus it can be seen that the intervention drugs and the included objects are different, it is reasonable that these clinical factors are no longer significantly related to tumor recurrence in our study.

The risk of recurrence in the incompleted instillation group was 3.57 times higher than that in the standard instillation group. Previous studies have shown that an immediate intravesical chemotherapy after TURBT reduced the recurrence rate of intermediate and low risk NMIBC [18, 19]. Nevertheless, more than half people in incompleted instillation group (18/ 32) did not undergo any intravesical instillation, which seemed to be the reason for the lowest 3-yr RFS.

Moreover, the Kaplan-Meier curve showed that the 3-yr and 5-yr estimated recurrence-free rate in the incompleted instillation group were both less than 20%, while those in the maintenance instillation group(> 13) were 80% and 65%, respectively(Fig. 1), which more accurately reflected the magnitude benefit for intravesical chemotherapy [20].

The benefit could be due to the ablative activity of the monthly instillation on small recurrent lesions [21]. Using a smoothed hazard function, Hinotsu et al. [22] reported that a peak of tumor recurrence after TUR of Ta/T1 bladder cancer was detected during the first 500 days after operation, and that a prophylactic effect was achieved during the same time. It was surmised that the peak hazard of recurrence at around 1.5 years could not be suppressed because of the instillations after only 8 weeks in the standard instillation group.

Finally, from the cut-off point of the ROC curve, maintaince instillation for 10–11 months seemed to be the optimal duration of chemotherapy in our scheme. The maintenance time was also within the range recommended by the AUA and EAU guidelines [12, 13].

Our research still had limitations. First, the ages of patients in the case group were relatively high. We set age limitation and evaluated the comorbidity to avoid this bias as far as possible. Second, this was a retrospective study with adherent limitation. A larger multi-center cohort study was needed to verify the threshold selected by this study indifferent races. Third, side effects such as cystitis and hematuria were not recorded.These side effects or other reasons such as poor compliance might lead to less instillations, which was likely to bias the results. Despite the above limitations, we accurately recorded the instillation frequency and recurrence, which was essential for the reliability of the study. 

## Conclusions

In summary, our study revealed that bladder instillation chemotherapy with a maintenance regimen more than 6 months confers a superior benefit of tumor recurrence in intermediate-risk NMIBC. Furthermore, maintenance chemotherapy for 10.5 months seemed to be the balance between economic cost and treatment benefits for intermediate risk NMIBC patients. Our findings provided new clues to developing the optimal instillation scheme and valuable experience to the precise medical treatment of intermediate risk NMIBC patients.

## Data Availability

The datasets generated and/or analysed during the current study are not publicly available due the results are part of the original data, which has not been fully published, but are available from the corresponding author on reasonable request.
